# What Is an Attractive Body? Using an Interactive 3D Program to Create the Ideal Body for You and Your Partner

**DOI:** 10.1371/journal.pone.0050601

**Published:** 2012-11-29

**Authors:** Kara L. Crossley, Piers L. Cornelissen, Martin J. Tovée

**Affiliations:** 1 Institute of Neuroscience, Newcastle University, United Kingdom; 2 Department of Psychology, University of Northumbria, United Kingdom; University of Melbourne, Australia

## Abstract

What is the ideal body size and shape that we want for ourselves and our partners? What are the important physical features in this ideal? And do both genders agree on what is an attractive body? To answer these questions we used a 3D interactive software system which allows our participants to produce a photorealistic, virtual male or female body. Forty female and forty male heterosexual Caucasian observers (females mean age 19.10 years, s.d. 1.01; 40 males mean age 19.84, s.d. 1.66) set their own ideal size and shape, and the size and shape of their ideal partner using the DAZ studio image manipulation programme. In this programme the shape and size of a 3D body can be altered along 94 independent dimensions, allowing each participant to create the exact size and shape of the body they want. The volume (and thus the weight assuming a standard density) and the circumference of the bust, waist and hips of these 3D models can then be measured. The ideal female body set by women (BMI = 18.9, WHR = 0.70, WCR = 0.67) was very similar to the ideal partner set by men, particularly in their BMI (BMI = 18.8, WHR = 0.73, WCR = 0.69). This was a lower BMI than the actual BMI of 39 of the 40 women. The ideal male body set by the men (BMI = 25.9, WHR = 0.87, WCR = 0.74) was very similar to the ideal partner set by the women (BMI = 24.5, WHR = 0.86, WCR = 0.77). This was a lower BMI than the actual BMI of roughly half of the men and a higher BMI than the other half. The results suggest a consistent preference for an ideal male and female body size and shape across both genders. The results also suggest that both BMI and torso shape are important components for the creation of the ideal body.

## Introduction

What makes a human body attractive to the opposite sex? In evolutionary psychology terms it is a judgment of a potential partner’s health and reproductive potential [1], [2]. In this context it is important that we are able to detect and accurately assess the physical cues that indicate that one individual is more attractive (i.e., fitter and with a better reproductive potential) than another, and then use these cues to choose the partner who is most likely to enhance our chances of successful reproduction [1]–[3]. As a result there should be a strong selective pressure to detect and accurately evaluate reliable cues to health and fertility in potential partners. However, there remains considerable debate over which cues are used to judge human physical attractiveness, their relative importance and whether these cues differ between men and women.

Previous studies that have attempted to define the importance of these physical cues have had a significant limitation. These studies have used line-drawings, photographs and, more rarely, video clips and 3D laser scans as test stimuli [4]–[18]. Typically, observers are asked to rate a set of images that vary on a number of anthropometric dimensions. However, these studies all suffer from the same intrinsic methodological limitation that they require their participants to rate bodies from the limited set of alternatives presented to them. Unfortunately, the ideal combination of features may not be included in the set of images with which they are presented. Thus, their apparent preference may actually be for a suboptimal body size and shape. To try and overcome this problem some researchers have presented participants with silhouettes or photographs in interactive computer programmes which allows the simple alteration of certain body features [19]–[21]. However, these techniques are obviously limited in the range of shape changes that can be made and the realism of the bodies produced. Additionally, the 2D representation of the bodies limits what can be seen of the change in the physical dimensions produced by the programme. It can be difficult to extrapolate from a 2D representation of a body to its 3D shape [22].

To overcome these important methodological limitations, we have used an interactive 3D software programme to determine male and female participants’ perceptions of their ideal body and their ideal partners’ body size and shape. The participants could alter the virtual 3D image of the body in more than 90 independent dimensions allowing very subtle changes in body shape. The body could be rotated through 360° to allow our participants to examine the body from different viewpoints. The scaled volume of these 3D models can then be measured and, assuming they have a standard body density, their body weight can then be estimated. Additionally, the scaled circumference of the chest, waist and hips of each body can be measured to allow the waist-to-hip ratio (WHR) and the waist-to-chest ratio (WCR) to be calculated. By taking anthropometric measures from all our participants, we can determine whether the participants’ own physical dimensions influence their choice of their own ideal body. This morphing technique allows us to answer two key questions:

*What is the ideal body size and shape?* For women, several studies have suggested that the ideal body is based on a curvaceous body, with a curvy lower torso (indexed by the WHR) but also a curvaceous upper body (WCR) [11], [13], [23]. Set against this, is an alternative hypothesis which postulates that the primary predictor of female attractiveness is overall body fat (usually measured as the Body Mass Index or BMI) [4], [12], [16], [17]. Changes in BMI have a strong impact on both health [24], [25] and reproductive potential [26]–[28], and a low WHR and WCR (i.e., a curvaceous body) is believed to correspond to the optimal fat distribution for high fertility [23], [29]. So there are clear reasons why both these features might impact on attractiveness judgements.

A similar difference of opinion exists for what is the main determinant of male attractiveness. Some studies assert that upper body shape (a broad upper body and a narrow waist, the classic V-shape) is the primary predictor of attractiveness, whereas others point to BMI as the key feature [5], [8], [10], [30]. It has been suggested that this v-shaped torso represents a muscular, strong body type that would be an advantage in our ancestral environment and therefore be sexually selected [10], [31]. BMI is an important predictor of male health and mortality [32], [33], and a narrow waist circumference is also important in long-term health and so should also be associated with a low WHR [34], [35].

By asking both men and women to set their ideal bodies we can determine which features they change and how their ideal body differs from their actual bodies. We can see whether they change shape or size or both.

*Do men and women share body ideals?* A number of studies have suggested a difference between the genders for the ideal body size and shape of a particular gender (for example, men may prefer a more curvaceous, heavier female body than women think they do) [36]–[38] and eye-tracking studies have suggested significantly different patterns of eye-movements between the genders when assessing female attractiveness [39]. However, mate selection theory predicts that an individual will have a very precise and accurate idea of what the opposite sex find attractive [3]. This allows them to judge their own relative value, with respect to their peer group, and match this value with the value of a prospective mate. So mate selection theory predicts that there will not be any difference between men and women in their ideals for both genders. There is some evidence to support this hypothesis in rating studies which have suggested the same ideals are held by both genders [8], [40], [41]. Our technique will allow us to accurately determine whether there are gender differences in body preferences, even if they are comparatively subtle and would not be detected in the choice between bodies within an image set. However large the image set, it cannot provide a continuous smooth change along all feature dimensions and so can only provide a comparatively coarse grained assessment of attractiveness ideals.

## Materials and Methods

### Ethics Statement

The study was reviewed and approved by the School of Psychology Ethics Committee of Newcastle University.

### Participants

A total of 80 heterosexual Caucasian undergraduate students aged 18–21 (40 females mean age 19.10 years, s.d. 1.01; 40 males mean age 19.84, s.d. 1.66) were recruited from Newcastle and Northumbria Universities. Participation was voluntary. However some students gained course credit. All participants gave informed consent and the aims and procedure of the study were explained beforehand. None of the students had previous experience with using the software.

### Protocol

**Figure 1:**
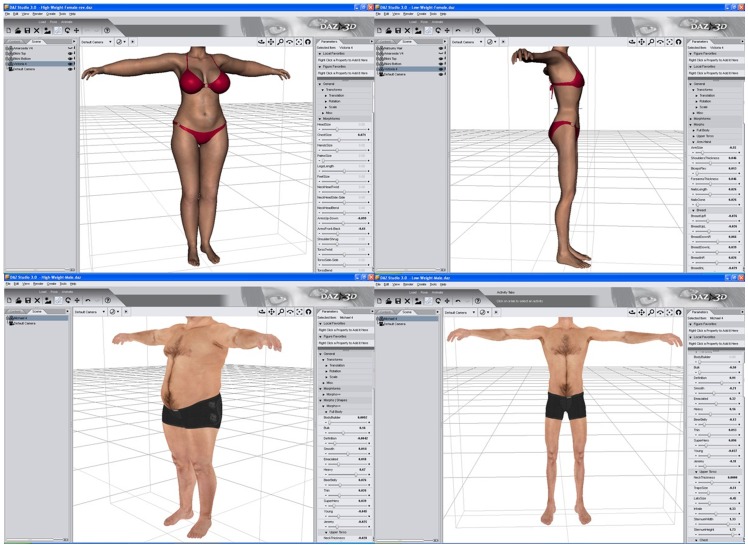
An example of the Daz3D interface, with examples of male and female bodies created in the software package. The bodies are displayed in slightly different viewing angles, and each body could be rotated though the whole 360°. Along the right of the picture are some of the 94 sliders which allowed different parts of the body to be independently altered.

The participants used a 3D modelling software package (Daz Studio 3.1 from Daz3d.com) which allows the adjustment of photo-realistic male and female 3D models on a flat panel screen in order to modify different aspects of the body’s features (see fig 1). The female 3D model used was Victoria 4.2 and the male model was Michael 4.0. The program allows the body to be rotated to allow a 360° view of the model. Along one side of the model is a set of 94 graphic sliders with which different aspects of individual body parts can be altered (using the ‘Body morphs’ and ‘Body morphs++’ add-on packages from Daz3D). When the slider is adjusted, the model simultaneously changes, providing immediate visual feedback. Sliders could be adjusted as many times as necessary and no time limit was set, so the participants could take as much time as they wished to satisfy themselves that the model was as accurate a representation as possible. The model was positioned so that the head was not visible and did not play a role in the judgements.

Each participant created a total of four 3D bodies; two that represented their ideal body and two that represented their ideal partner’s body. In each of the two conditions, the participants began with a ‘heavy’ body and then a ‘thin’ body, or vice versa. The order was counterbalanced between participants. The two estimates were averaged to render a final model. The use of fat/thin bodies as a starting point was to reduce potential anchor effects which might have occurred if participants had just begun by adjusting a normal weight body. The female “thin” body had a BMI of 14.9 and the “large” body had a BMI of 26.6. The male “thin” body had a BMI of 16.5 and the “large” body had a BMI of 37.7.

All the participants were tested on the same PC in the Body Image Lab at the Institute of Neuroscience. Participants were asked to adjust the sliders until they were satisfied that the model looked like their ideal body and then they were asked to produce their ideal partner’s body. No time limit was placed upon them. Although there are 94 sliders, many of them are used for comparatively subtle adjustments to features such as the length of the ring finger on the left hand, and were not used. We ourselves had not altered these minor features in the “heavy” and “thin” bodies and we had left them at the default setting. Instead, most participants used a core set of sliders (mean 36.2 sliders, s.d. 7.8) which changed features, such as stomach depth and hip width.

After completion, a set of anthropometric measures were taken from the participants by the lead author (K.L.C.). Height was measured using the Marsden/Invicta Free Standing Height Measure and weight was measured using the Weight Watchers 8944U Heavy Duty Body Fat Analyser Scale. Using a standard tape measure, the waist and hip circumferences were measured, along with bust and under-bust circumferences if female, and chest circumference if male, following the protocols outlined in the Health Survey for England [42].

### The 3D Body Analysis

The final 3D models were exported from Daz Studio, once clothing had been removed, and reopened in 3ds Max (autodesk.com), where they were set either to the height of the participant (for their own ‘ideal’) or to the height of the average British man (1.78 m) or woman (1.64 m) (for ‘ideal partner’). First, the volumes of the 3D models were calculated by the software, scaling the body volume relative to the body height entered by the experimenter. Once the volumes were known, the weights of the models were estimated by multiplying their volumes by the density of either the average young adult female body (1.04 g/cm^3^) or the average young adult male body (1.06 g/cm^3^) [43], [44]. Finally, the BMI of each model was calculated as its weight (kg) divided by its height (m) squared.

Next, 3ds Max was used to slice through each model at predetermined points along its length to measure the circumference of the bodies at the chest, waist and hips in male models, and the bust, under-bust, waist and hips in female models. The software scaled the circumferences (measured in cm) to the dimensions that the bodies would have if they were real. However, the circumference measures generated by 3ds Max for the hips in male bodies and bust and hips in female bodies tend to be larger than the same measurements taken from real bodies. This is because 3ds max calculates the path length around - each slice which includes, for example, the cleft in the bust or buttocks. In comparison, a tape measure looped around the bust or hips will straddle these gaps, and so will produce a shorter distance. To compensate for these effects, we screen grabbed the cross-sectional slices of the bust or hips in 3ds Max and imported them into ImageJ (http://rsbweb.nih.gov/ij/). There we used the lasso drawing tools to replicate the path that a tape measure would take when placed around the bust or buttocks, and used the measurement tools to calculate the path length which better reflected a real world measurement with a tape measure.

### Test-Retest Reliability

A potential weakness of this methodology is the question of whether the participants can reliably manipulate the software controls to produce the body size and shape that they want. To answer this question we ran a test-retest experiment in which participants were asked to repeat the modelling task. We asked 15 Caucasian female participants (average age 22.57, s.d. 2.76) and 15 Caucasian male participants (average age 23.21, s.d. 2.84) to set their ideal body size and shape using the same methodology as described above. They then repeated the same tasks the following day. The Pearson’s correlation between the BMI values of the models that participants set on the two days was highly statistically significant (r = 0.99, p<.001) and a paired-samples t-test of the bodies’ BMIs showed no significant differences between the settings for the bodies on the two days (paired T-test: t(25) = 1.69, p = .103).

## Results

In this results section we first show that that there are significant differences in size and shape between the actual bodies of the participants and their ideals. We then show that these ideal bodies differ from the expected shape of real bodies of the same BMI, implying an explicit choice for specific sizes *and* shapes in their ideal bodies. Finally, we show that the ideal size and shape for both a male and a female body is shared by both our male and female participants (i.e. there is no gender based difference on what constitutes an attractive male or female body).

### Comparisons of Participants’ Actual BMI versus Ideal BMI

**Figure 2:**
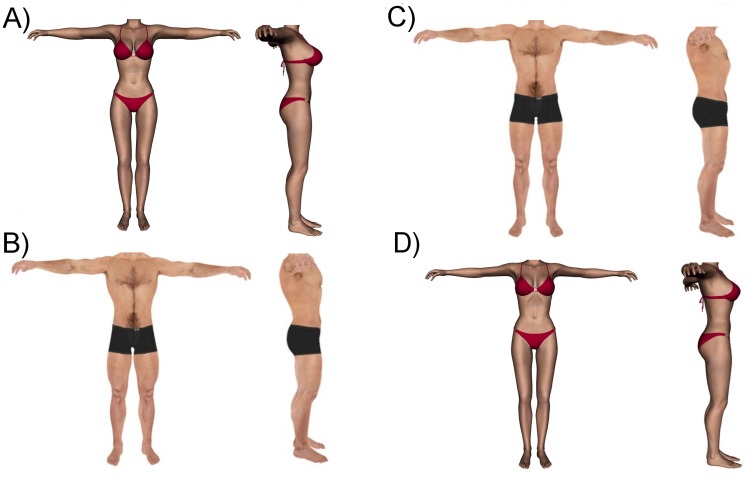
**Figure 2** shows examples of the bodies set by the female participants (A & B) and the male participants (C & D). Body A and C are the ideal female bodies set by the female and male participants respectively and Body B and D is the ideal male body set by the female and male participants respectively.

A summary of the anthropometric data from the participants’ actual and ideal bodies are shown in table 1 and examples of the ideal bodies are shown in figure 2. A comparison of the BMI values for the male participants’ actual body and their male ideal body showed a significant increase in the BMI of the ideal body (paired T-test: t(39) = −2.26, p = .029; effect size r = 0.34; power to detect at two-sided alpha of 0.05 = 0.59) [45]. This difference is also significant for the female participants who showed a significant reduction in the BMI of their ideal body (paired T-test: t(39) = 7.49, p<.0001; effect size r = 0.77; power to detect at two-sided alpha of 0.05>.99 ).

**Table 1:** Table 1 summarises the anthropometric measures taken from the male and female bodies in this study.

	BMI	Bust/Chest	Under Bust	Waist		Hips	WCR	WHR
**Female Actual Body**								
Average	21.7	87.4	75.93	72.91	99.4		0.86	0.73
SD	2.07	5.17	5.6	5.48	5.36		0.2	0.19
**Female’s Ideal Body**								
Average	18.85	93.97	68.33	61.12	87.89		0.67	0.70
SD	1.75	8.24	4.06	3.38	6.52		0.09	0.04
**Male’s Ideal Female Body**								
Average	18.82	90.02	69.2	61.95	84.82		0.69	0.73
SD	1.56	4.73	5.79	5.79	4.92		0.05	0.04
**Male Actual Body**								
Average	24.54	97.74	–	86.12	98.76		0.88	0.87
SD	3.38	9.21	–	9.47	7.93		0.04	0.06
**Male’s Ideal Body**								
Average	25.86	111.26	–	82.00	91.17		0.74	0.87
SD	3.95	9.44	–	9.17	9.59		0.05	0.04
**Female’s Ideal Male Body**								
Average	24.46	104.16	–	80.57	90.81		0.77	0.86
SD	2.9	7.43	–	7.22	7.15		0.05	0.03

### General Patterns of Shape Change Estimated by WHR and WCR Comparing Male with Female Actual versus Ideal Bodies

**Figure 3:**
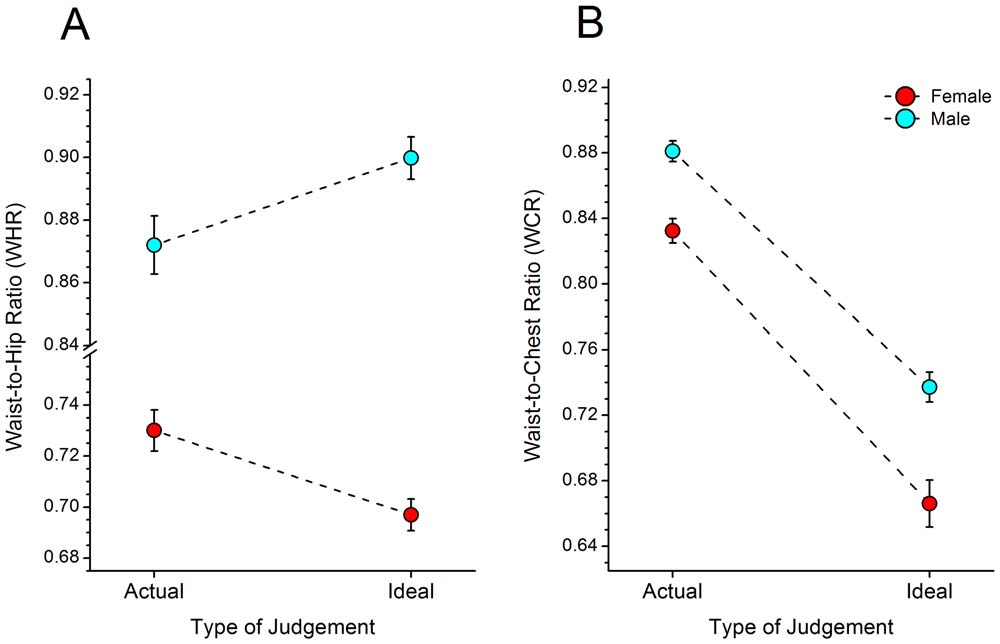
Fig. 3A shows a plot of the average actual and ideal WHRs of male and female observers. Males appear to prefer a more tubular shape in their lower torso (as indexed by a higher WHR) as their ideal. In comparison, females appear to desire a curvier lower torso shape (with a lower WHR values) for their ideal. Fig. 3B shows an equivalent plot for WCR. Both male and female participants preferred a lower WCR (more curvaceous) in their ideal than they actually possessed.

Fig. 3A shows a plot of the actual and ideal WHRs of male and female observers. It shows that WHRs are generally larger for male bodies than for female bodies. Moreover, males appear to prefer a more tubular shape in their lower torso, indexed by a higher WHR, as their ideal. In comparison, females appear to desire a curvier lower torso shape, as indexed by lower WHR values for their ideal. Fig. 3B shows an equivalent plot for WCR. Again, male observers have higher WCR’s overall compared to females. However, both genders appear to desire larger circumference chests than waists by about the same proportion in their ideal figures.

To quantify these effects we computed a between-subjects (i.e. gender: male versus female) ANOVA and a within-subjects (i.e. condition: actual versus ideal) mixed ANOVA separately for WHR and WCR.

For WHR, we found a statistically significant main effect of gender (F_1,117_ = 506.48, p<.0001), but not of condition (F_1,117_ = 0.26, p = .613). The interaction between gender and condition was statistically significant (F_1,117_ = 17.34, p<.0001). In order to identify which differences contributed to the interaction term, we computed post-hoc differences of least square means using the Tukey-Kramer correction to compensate for multiple statistical comparisons. We found that the female participants’ ideal WHRs were significantly less than their actual WHRs (t_117_ = 3.30, p = .0069; r = 0.29) and that the males’ ideal WHRs were significantly higher than their actual WHRs (t_117_ = −2.59, p = .05; r = 0.23).

For WCR, we found a statistically significant main effect of gender (F_1, 156_ = 37.15, p<.0001) and condition (F_1, 156_ = 249.13, p<.0001). However, the interaction between gender and condition was not statistically significant (F_1,156_ = 1.31, p = .254). We computed post-hoc differences of least square means using the Tukey-Kramer correction to compensate for multiple statistical comparisons and found that females’ and males’ ideal WCRs were significantly lower than their actual WCRs (t_156_ = 11.97, p<.0001; r = 0.69 and t_156_ = 10.35, p<.0001; r = 0.64 respectively).

### The Non-Linear Co-Variation of Body Mass and Shape

**Figure 4:**
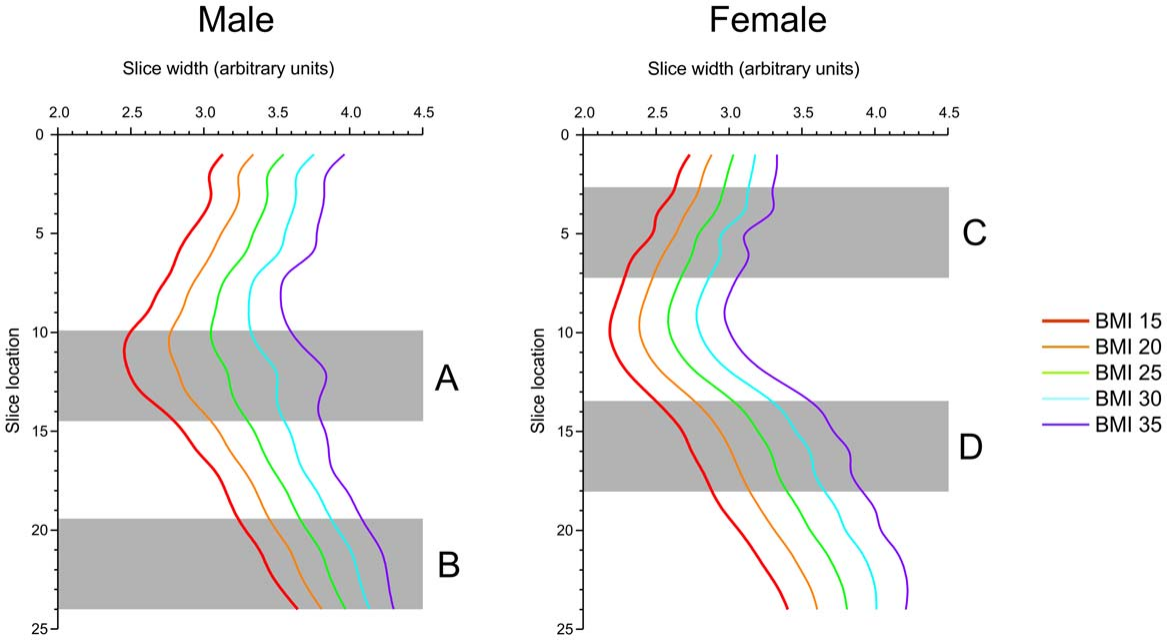
Plots of the width of the right side of the torso, starting from the midline, for the average male and female bodies at five different BMI levels. The plots illustrate that increasing BMI is associated not only with a generalized increase in torso width, reflected by the systematic separation of one profile from the next, but also with a non-linear component to the change in body shape. This non-linear component is illustrated by the male torso outline in sub-regions A (near the waist) and B (the lower hip). In region A, as BMI increases from 15 to 35, the contour of the waist changes from convex to concave and in region B, the slope of the line from lower to higher hip slices becomes less and less steep. There are similar non-linear shape changes in the female torso in sub-regions C (the upper chest) and D (upper hip).

Our analysis suggests that the ideal body size and shape of both the male’s and female’s ideals differs from the corresponding actual bodies. However, a possible confound is that in real life, body shape and body size tend to co-vary in a non-linear way (i.e. a body with a particular BMI will have a particular shape), with different parts of the body changing size at different rates with changing BMI. We have already illustrated this relationship in women’s bodies in several previous studies [17], [46], [47] and we can illustrate this co-variation here in male and female bodies by plotting the torso width of a set of 122 young Caucasian men (average age 27.4, s.d. 11.9) and 60 young Caucasian women (average age 26.1 years, s.d. 6.7) who agreed to be photographed to provide stimuli for a number of studies of physical attractiveness (see [8], [46], [48]). The widths of 31 slices taken through the torso of 2D frontal images of the participants were obtained, along with their respective BMIs (see Fig. 4). The location for each slice was standardized across participants by equally dividing the distance between fixed anatomical landmarks (the acromio-clavicular joint and the perineum) into 30 equal partitions. This is illustrated in Fig 4, which shows a plot of the width of the right side of the torso, starting from the midline, for the average male and female body at five different BMI levels.

A simple regression can then be used to estimate the relationship between each slice width and BMI. The key feature to appreciate about fig. 4 is that increasing BMI is associated not only with a generalized increase in torso width, reflected by the systematic separation of one profile from the next, but also with a non-linear component to the change in body shape. This non-linear component is illustrated by considering, for example, the male torso outline in sub-regions A (near the waist) and B (the lower hip) in Fig. 4. In region A, as BMI increases from 15 to 35, the contour of the waist changes from convex to concave. Over the same BMI rage, the slope of the line from lower to higher hip slices becomes less and less steep. Therefore, it is clear that by selecting an ideal body with a different BMI, participants are implicitly selecting a complex change in the shape of the ideal body. There are similar non-linear shape changes in the female torso that can be seen in sub-regions C (the upper chest) and D (upper hip) of Fig. 4.

In the current study we seek to answer the question of how different are people’s own ideal body shapes compared to the shape they currently have, as well as the ideal body they would seek in a partner. The complex shape changes illustrated in Fig. 4 that occur as a result of changing BMI demonstrate that this question needs to be carefully refined. It could be that when people pick an ideal body shape, what they are really doing is picking a body which for them represents a body with an ideal BMI. Not only is this choice necessarily associated with a change in the width of the body, but also there are additional shape changes caused by the fact that fat is not deposited equally around the body. Therefore, in addition to any width changes represented in the ideal body, there are also non-linear shape changes associated with a change in BMI as illustrated in Fig. 4. An alternative possibility, when people are asked to pick an ideal body shape, is that they may choose a shape which goes beyond any changes attributable to a change in BMI alone, including the linear and non-linear components. Therefore, in the analysis that follows, we address this confounding problem directly.

Since the BMI of both genders’ ideals is different from their actual BMI, we can calculate what proportion of the change in torso shape of their ideal body is attributable just to the change in BMI alone. In other words we can predict the component of shape change in the ideal which is predicted by the BMI of the ideal body shape selected. We can then compute the difference between the bust/chest, under-bust, waist and hip circumferences of the ideal image and the equivalent circumferences computed on the basis of the BMI of the ideal and then ask whether, on average, these are significantly different from zero. If this population of differences is not significantly different from zero, this suggests that the shape of the body that participants choose as their ideal is no different from merely choosing a higher or lower BMI. However, if the population of differences in circumferences is significantly different from zero, this means that the shape of the bodies that participants choose as their ideal is different from what they would achieve by merely selecting a higher or lower BMI.

The regression analyses to estimate the BMI shape change effect are based on circumference measures taken from 120 male and 120 female volunteers. The females were measured at bust, under-bust, waist and hips and the males at chest, waist and hips. The average age of the female volunteers was 20.3 years s.d. 3.5 and the average age of the male volunteers was 20.7 years s.d. 2.1. For each gender, we computed separately the regression between BMI and chest/bust, under bust waist and hip respectively, and then used these regression equations to estimate the expected circumferences in the ideal bodies chosen, based purely on their BMI.

### Are the Circumferences of Ideal Male Bodies Different from those Expected from their BMIs?

Table 2 below shows the descriptive statistics for the difference between the circumferences of the 3D model settings for the ideal male body shapes set by both male and female participants and those predicted from the BMI of the ideal models. Table 2 shows that both male and female participants set ideal body shapes which have chest circumferences substantially larger than the chest circumference attributable to the lighter BMI ideal set in section above. Moreover, the commensurate waist and hip circumferences are both substantially smaller than the values predicted on the basis of the ideal BMI that was selected in each case.

**Table 2:** Summary of the comparison between the ideal male body set by the participants and the body predicted by the BMI.

Group	Body Slice	Average Difference in Circumference (cm)	t-test value	p value	r value	Power
**Male’s ideal male body**	Chest	11.04 (0.86)	12.76	<.0001	0.90	>.99
	Waist	−12.92 (0.69)	−18.67	<.0001	0.95	>.99
	Hips	−9.64 (0.59)	−16.46	<.0001	0.93	>.99
**Female’s ideal** **male body**	Chest	6.93 (0.92)	7.52	<.0001	0.77	>.99
	Waist	−10.99 (0.52)	−21.08	<.0001	0.96	>.99
	Hips	−7.60 (0.46)	−16.55	<.0001	0.94	>.99

The difference in the slice circumferences from the two bodies are shown along with the standard error in brackets. The DF for the t-test was 39.

To further explore this result we carried out t-tests for each set of circumferences (i.e. chest, waist, hips) for the populations of differences (see table 2), where the null hypothesis was a mean of zero. All are statistically significant at p<.05, even after applying a Bonferroni correction for multiple comparisons.

### Are there Differences in the Circumferences of the Ideal Male Bodies set by the Male and Female Participants?

The results from the T-tests of location above show that, the average shape of the ideal female bodies set by male and female participants differs significantly from the shape that would be predicted based solely on the BMI of the ideals. Next, we test whether the shapes of these ideals differ when comparing the settings made by male versus female participants. To address this question, we used a 2-factor, repeated-measures GLMM, where factor 1 was the gender of the participant (male, female) and factor 2 was the circumference (chest, waist and hip). There was no main effect of gender (F_1,234_ = 0.01, P = .938). The main effect of circumference was significant (F_2,234_ = 523.42, p<.0001) as was the interaction between gender and circumference (F_2,234_ = 12.78, p<.0001). To determine which individual ideal shape measures differed between male and female participants, we calculated post-hoc differences of least square means using the Tukey-Kramer correction to compensate for multiple statistical comparisons. The difference between male and female settings of chest circumference was statistically significant (p<.0001), whereas the differences for waist and hip were not.

### Is the Ideal Male Body Different in Size and Shape for the Male and Female Participants?

An independent t-test shows that the ideal male BMI set by the female participants is not significantly different from that set by the male participants (t(78) = 1.81, p = 0.074; effect size r = 0.20; power to detect at two-sided alpha of 0.05 = 0.44). The WHR of the two bodies were also not significantly different: t(78) = 1.43, p = .229; effect size r = 0.16; power to detect at two-sided alpha of 0.05 = 0.20), but WCR was significantly different (t(78) =  −3.09, p = .003; effect size r = 0.33; power to detect at two-sided alpha of 0.05 = 0.67).

### Are the Circumferences of Ideal Female Bodies different from those Expected from their BMIs?

Table 3 below shows the descriptive statistics for the difference between the circumferences of the 3D model settings for the ideal female body shapes set by both male and female participants and those predicted from the BMI of the ideal models. Table 3 shows that both male and female participants set ideal body shapes which have bust circumferences substantially larger than the bust circumference attributable to the lighter BMI ideal set above. Moreover, the commensurate under-bust, waist and hip circumferences are substantially smaller than the values predicted on the basis of the ideal BMI that was selected in each case.

**Table 3:** Summary of the comparison between the ideal female body set by the participants and the body predicted by the BMI.

Group	Body Slice	Average Difference in Circumference (cm)	t-test value	p value	r value	Power
**Female’s ideal** **Female body**	Bust	10.78 (2.97)	3.62	.0008	0.50	>.94
	Under Bust	−3.73 (0.60)	−6.19	<0.0001	0.70	>.99
	Waist	−6.43 (0.47)	−13.68	<.0001	0.91	>.99
	Hips	−0.63 (0.77)	−0.81	.42	0.13	.12
**Male’s ideal** **Female body**	Bust	6.88 (0.81)	8.46	<.0001	0.80	>.99
	Under Bust	−2.81 (0.81)	−3.48	.001	0.50	.92
	Waist	−5.53 (0.48)	−11.56	<.0001	0.88	>.99
	Hips	−3.64 (0.57)	−6.40	<.0001	0.72	>.92

The difference in the slice circumferences from the two bodies are shown along with the standard error in brackets. The DF for the t-test was 39.

T-tests of location for the populations of differences, where the null hypothesis was a mean of zero, are all statistically significant at p<.05, even after applying a Bonferroni correction for multiple comparisons, with the exception of female settings for the hip circumference.

### Are there Differences in the Circumferences of the Ideal Female bodies set by the Male and Female Participants?

The results from the T-tests of location above show that the average shape of the ideal female bodies set by male and female participants differs significantly from the shape that would be predicted based solely on the BMI of the ideals. Next, we test whether these ideal body shapes differ when comparing the settings made by male versus female participants. As before, to address this question, we used a 2-factor repeated measures GLMM, where factor 1 was the gender of the participant (male, female) and factor 2 was the circumference (bust, under-bust, waist and hip). There was no main effect of gender (F_1,78_ = 1.67, P = .201). The main effect of circumference was significant (F_3,234_ = 63.68, p<.0001), but there was no significant interaction between gender and circumference (F_3,234_ = 2.43, p = .066).

### Is the Ideal Female Body Different in Size and Shape for the Male and Female Participants?

An independent t-test shows that the ideal female BMI set by the female participants is not significantly different from the ideal female BMI set by the male participants (t(78) = 0.09, p = 0.93; effect size r = 0.01; power to detect at two-sided alpha of 0.05 = 0.05). The WBR of the two bodies were also not significantly different (t(78) =  −3.64, p<.001; effect size r = 0.38; power to detect at two-sided alpha of 0.05 = 0.91), but WHR was significantly different (t(78) =  −3.64, p<.001; effect size r = 0.38; power to detect at two-sided alpha of 0.05 = 0.91).

## Discussion

### What is the Ideal Female Body Size and Shape?

Both male and female participants created an ideal body that was significantly different in body size relative to their own. The female participants significantly reduced the body size and the male participants increased it. Although some studies have suggested BMI is the primary predictor of female attractiveness and that shape is of marginal importance (e.g. [4], [12], [17], [46], this study suggests that body shape is a significant factor, at least with respect to the perception and creation of ideals. Shape and body mass co-vary (e.g. [17], [47], [49], [50], but by controlling for the expected changes which occur with changing BMI, we show that both male and female participants nevertheless produce ideals with a specific shape which is independent of the ideal’s BMI.

The female participants’ ideal female body has a BMI which is significantly lower than their actual BMI. Consistent with this lowered BMI, there is a general narrowing of the torso, with the hips, waist and chest (excluding the bust) reducing in circumference (i.e. the volume of the body is reduced). The actual BMI values of the female participants all fall within the normal BMI range (18.5-24.9), with the majority around the middle part of this scale [51], [52]. While their ideal female body is also just within the normal range, it is only just above the underweight category. However, this is consistent with previous studies in which photographs of women’s bodies have been rated for attractiveness which have suggested an ideal BMI of as low as 18–20 for Western male and female observers [16], [40]. Only 1 of the 40 female participants wanted an ideal BMI above their actual BMI. This low ideal BMI is similar to the BMI reported for female models appearing in the media [53], [54], a result consistent with the hypothesis that low BMI women in the media influence body size preferences [55]–[58] and contribute to the high proportion of women who show dieting and weight loss behaviours even though they have a normal BMI [59]–[61]. The participants in our experiment are university students and are therefore a relatively young group who may be more sensitive to media influence on body ideals than older people (e.g. [62], [63]). However in previous attractiveness studies which have used participants with wide age ranges we have not found differences in their ideal size and shape [64] suggesting the findings in the current study are representative of the general population.

In contrast to the narrowing of the rest of the female body, the “ideal” bust increases in size (as indexed by bust circumference). Previous studies have linked relative bust size to circulating estrogen levels, with the suggestion that a large bust and a narrow waist should indicate high levels of estrogen and therefore be regarded as attractive [25]. A number of studies have suggested that female bodies with a larger bust are considered to be more attractive [20], [65] and breast augmentation is the most common cosmetic surgical procedure in the UK and US [66]. The large bust and low BMI set by both the male and female participants also reflects the size and shape of glamour models in men’s magazines which are often taken as a proxy for a cultural ideal of female beauty [53], [54].

The increase in bust size and narrowing of the torso between the female participants’ actual body and their ideal changes the upper body shape (as indexed by WCR and illustrated in figure 3). The female participants also narrow their hips as well as their waist, but because there is a relatively greater narrowing of the waist, the lower torso also increases in curvature (as indexed by WHR). There is less change in the WHR than in WCR, but this may be because the WHR of the participants’ actual body is already quite close to a value of 0.7 which has been suggested to be optimal for health and fertility and thus also for attractiveness [11], [13].

### What is the Ideal Male Body Size and Shape?

Unlike the female thin ideal body, the ideal male body is comparatively heavy, falling at the boundary of the normal to overweight categories of the BMI scale. However, these are not bodies that look over-weight, but instead are big and muscular. In fact, our calculation of their BMI is probably an under estimation, because we are assuming that the bodies have the average density for young men (i.e. the average balance of fat to muscle). As muscle is approximately 20% denser than fat, this would under-estimate the mass of a more muscular body such as the male ideals set in this experiment. This result is consistent with previous studies, which have suggested that muscularity (and the associated perception of dominance and strength) is the primary determinant of male attractiveness [8], [10], [30]. Whereas there is a tendency for women to diet to achieve their ideal body, young men are more likely to be influenced by magazines to build up a bigger, more muscular body [31], [66]. So although the male ideal body is heavier, the additional weight is muscle rather than fat. As discussed above, BMI is a measure of body weight scaled for height and not a direct measure of percentage body fat. Its use in epidemiological studies is due to its ease of administration. The ideal male body set by both male and female participants is lean with high muscle definition (which requires a percentage body fat below 9–12%). Our participants’ male ideal is both muscular and low in body fat.

The male participant’s ideal body shows an increase in chest circumference (relative to their actual body) and a reduction in the waist and hips to produce a V-shaped upper body. Previous studies have also suggested that men prefer a body that is more muscular than the one they actually possess [31], [67]–[69]. It is suggested that a v-shaped upper body is a key predictor of male attractiveness judgements because this indicates upper body strength [8], [10], [30], [31], [70]. By contrast, the ideal lower body is narrowed relative to the actual body making it less curvy and more straight-up and down. This is the opposite of what is found for the ideal female bodies.

### Do Men and Women Share Body Ideals?

The preferences for the ideal female body are broadly similar between the two genders. They both prefer the same low BMI and a relatively curvaceous body with WCR and WHR with values around 0.7. There is also general agreement between the genders on the ideal male body; this male ideal has a relatively large body with a V-shape upper torso and a narrow waist and hips. This is consistent with attractiveness rating studies which to show a strong correlation between male and female attractiveness ratings of male and female bodies (i.e. both genders seem to rate bodies of both genders the same way) [8], [17], [46], [48].This can be explained by mate selection theory which suggests that individuals will not only be able to judge the attractiveness of members of the opposite sex, but will also know their own attractiveness relative to other members of the same sex (i.e. their competitors) [3]. This information allows an individual to concentrate on potential partners of the same attractiveness as themselves, thus avoiding both unsuccessful courtship of a more attractive partner (potentially wasteful in time and resources) and accepting a less attractive partner (with a potentially negative impact on future reproductive success). Thus an individual must be able to assess bodies of their own gender using the same attractiveness criteria as the opposite sex, and by extension, must therefore have a good idea of the opposite gender’s ideal partner. So our female and male participants should share the same ideals for both male and female bodies.

An alternative explanation would be that the ideals are influenced by a common media environment which pushes them towards the same concept of the ideal body. However, there are subtle gender-specific differences in the media images seen in the magazines targeted at men and women. For the male body, magazines aimed at a male audience contain male models which are more muscular than those aimed at a female audience [31], [66]. For the female body, female models in women’s magazines are slimmer and have a smaller bust than female models in men’s magazines [53], [54]. This would suggest that there should be systematic differences between the ideals favoured by the two genders.

This is partially what we find here. The male body selected by the male participants is indeed more muscular than the ideal male body chosen by the female participants. However, in the case of the ideal female body both men and women prefer a female body with the same low BMI, but the female participants prefer a larger bust size than the male participants. This directly contradicts what would be expected from the size and shape of the female models in their respective gender-specific media; the men should prefer a heavier female body than the women and a larger bust.

Previous studies have focussed on body size in women’s bodies. These suggest that although women overestimate the level of female thinness desired by men (e.g. [36], [37], [71], [72]), when asked to simply rate images without reference to what they think men would find most attractive, women and men have the same ideal BMI for female attractiveness [4], [12], [22], [46], [48]. Our study asked the female participants what they thought was the ideal body size and shape, and if we had asked them to choose what they thought a man would choose we might have got a difference between this body and the male judgement of female ideal body size.

That still leaves the question of why the difference exists in male and female preferences for upper body shape; female participants prefer a larger bust in their ideal female body than men, and male participants prefer a larger chest in their ideal male body than women. This may be linked to within gender competition for status and prestige [31], [70]. Many forms of prestige and status competition are between members of the same gender. Such a competition could produce a runaway process in which a physical feature becomes increasingly exaggerated over time due to competition between same-gender individuals. As this is a within gender competition the possibility exists that these processes will lead to divergence between preferences of the two genders for a specific feature, such as muscularity in men or bust size in women [10], [31], [70].

An alternative socio-cultural explanation would emphasise how a culture-specific female ideal body size and shape potentially exerts a particularly strong influence on women’s concept of what they should aspire to [73], [74]. This ideal, which is impossible for most women to achieve, is suggested to lead to body dissatisfaction and potentially in some cases to eating disorders [73], [75]. Women who do not conform to this ideal are more likely to receive negative comments and discrimination [76], [77], which serves to condition the importance of physical appearance as part of their estimation of self-worth [78], [79]. In this context, the importance of physical appearance is potentially clearest to young women (such as our participants) who are more likely to be actively involved in the mate selection process. This reinforcement of the perfect female ideal could potentially lead to an exaggeration of the internal representation of some of the ideal physical features [79]–[81], such as bust size in our female participants relative to the males. A similar process may also explain the exaggeration of the upper body musculature of the male ideals by the male participants. The propagation of the highly musculature male ideal through gender specific magazines [31], [66] and its reinforcement in young men by experience of mate competition with other men [1], may promote an exaggerated idea of the ideal male body shape.

### Conclusions

The combination of the 3D morphing software and the regression analysis shows that the ideals for both genders have a specific body size (as indexed by BMI) and shape. For both sexes, the primary predictor of female beauty is a relatively low BMI combined with a relatively curvaceous body, whereas the features important for the male ideal are a slightly heavier, muscled body with a specific V-shaped upper body. Although, the results suggest a largely consistent preference for an ideal male and female body size and shape across both genders, but with subtle differences based on an own gender exaggeration of upper body shape.
